# Long non-coding RNA LINC00346 promotes pancreatic cancer growth and gemcitabine resistance by sponging miR-188-3p to derepress BRD4 expression

**DOI:** 10.1186/s13046-019-1055-9

**Published:** 2019-02-06

**Authors:** Weidong Shi, Chenyue Zhang, Zhouyu Ning, Yongqiang Hua, Ye Li, Lianyu Chen, Luming Liu, Zhen Chen, Zhiqiang Meng

**Affiliations:** 1 Department of Integrative Oncology, Fudan University Shanghai Cancer Center, 270 Dong An Road, Shanghai, 200032 China; 2 Department of Oncology, Shanghai Medical College, Fudan University, Shanghai, China; 3 Collaborative Innovation Center for Cancer Medicine, Fudan University Shanghai Cancer Center, Shanghai, China

**Keywords:** Gemcitabine, Growth, LINC00346, miR-188-3p, Pancreatic cancer

## Abstract

**Background:**

Long non-coding RNA LINC00346 has been recently suggested as a prognostic marker in pancreatic cancer. However, its biological function in pancreatic cancer has not yet been determined. In this study, we attempted to ascertain the role of LINC00346 in regulating the aggressiveness of pancreatic cancer.

**Methods:**

The effects of overexpression and knockdown of LINC00346 on the proliferation, cell cycle progression, apoptosis, and gemcitabine resistance were investigated. Bioinformatic analysis, luciferase reporter assay, and RNA immunoprecipitation assay were performed to search for potential microRNAs (miRs) that can interact with LINC00346.

**Results:**

Overexpression of LINC00346 significantly enhanced the proliferation, colony formation, and tumorigenesis of pancreatic cancer cells. Conversely, knockdown of LINC00346 suppressed pancreatic cancer cell proliferation and caused a cell-cycle arrest at the G2/M-phase. Depletion of LINC00346 also enhanced gemcitabine sensitivity in pancreatic cancer cells both in vitro and in vivo. Mechanistic investigation revealed that LINC00346 acted as a sponge for miR-188-3p and blocked the repression of BRD4 by miR-188-3p in pancreatic cancer cells. Clinical evidence indicated a negative correlation between LINC00346 and miR-188-3p in pancreatic cancer specimens. Rescue experiments showed that LINC00346 attenuated the growth-suppressing and chemosensitizing effects of miR-188-3p on pancreatic cancer cells. In addition, silencing of BRD4 significantly inhibited LINC00346-induced pancreatic cancer cell proliferation and colony formation.

**Conclusions:**

LINC00346 shows the ability to promote pancreatic cancer growth and gemcitabine resistance, which is in part mediated by antagonization of miR-188-3p and induction of BRD4. Targeting LINC00346 may improve gemcitabine-based therapeutic efficacy.

**Electronic supplementary material:**

The online version of this article (10.1186/s13046-019-1055-9) contains supplementary material, which is available to authorized users.

## Introduction

Pancreatic cancer is one of the most lethal malignancies, with a median overall survival of 6 months and 5-year survival rate of less than 5% [1, 2]. The highly metastatic potential of tumor cells is an important reason causing the dismal prognosis in pancreatic cancer [3]. Gemcitabine, a deoxycytidine analog, is currently used as the first-line chemotherapy for metastatic pancreatic cancer [4, 5]. However, the therapeutic efficacy of gemcitabine is limited by intrinsic and acquired drug resistance [6]. Understanding the molecular mechanism governing gemcitabine resistance is of significance for improvement of therapeutic strategies against pancreatic cancer.

Long non-coding RNAs (lncRNAs) comprise a class of non-coding RNA transcripts of longer than 200 bp and are widely implicated in gene regulation [7]. LncRNAs play an important role in tumor progression and drug resistance [7, 8]. They can function as competing endogenous RNAs (ceRNAs) to sponge microRNAs (miRs), thus derepressing multiple target genes [9]. For instance, lncRNA XIST was reported to enhance pancreatic cancer growth by interacting with miR-133a [10]. Similarly, Linc-ROR was found to induce gemcitabine resistance in pancreatic cancer cells through regulation of the miR-124/PTBP1/PKM2 axis [11]. There is evidence that the lncRNA LINC00346 is upregulated in non-small cell lung cancer and bladder cancer and exerts oncogenic effects on the 2 cancer types [12, 13]. A recent study has showed that LINC00346 is overexpressed and serves as a prognostic marker in pancreatic cancer [14]. Despite these clinical observations, little is known regarding the function of LINC00346 in pancreatic cancer.

Bromodomain-containing protein 4 (BRD4) belongs to the bromodomain and extra terminal domain (BET) family that participates in epigenetic regulation of gene expression [15]. BRD4 has been found to modulate multiple aspects of tumor biology, including proliferation, migration, invasion, and survival. [16, 17] Li et al suggest that BRD4 is required for DNA repair and its inhibition exerts antitumor effects on multiple cancer types [17]. Andrews et al demonstrated that dual inhibition of BRD4 and PI3K remarkably suppresses cancer cell growth and metastasis [18]. Increased expression of BRD4 is noted in pancreatic cancer, and targeted reduction of BRD4 leads to inhibition of cancer growth [19]. Wang et al reported that BRD4 silencing enhances the chemosensitivity of pancreatic cancer cells to gemcitabine [20]. These studies suggest BRD4 as a promising therapeutic target for pancreatic cancer.

In this study, we aimed to explore the biological role of LINC00346 in regulating the malignant phenotypes of pancreatic cancer cells. Mechanistic analysis revealed that the enhancement of BRD4 contributes to the oncogenic activities of LINC00346.

## Materials and methods

### Cell culture

Human pancreatic cancer cell lines (PANC-1, MIA PaCa-2, Capan-1, and BxPC-3) were purchased from the American Type Culture Collection (ATCC, Manassas, VA, USA). HPDE6c7 pancreatic epithelial cells and 293 T cells were obtained from Jiangnin Biotech. Co. Ltd. (Shanghai, China). Cells were maintained in RPMI 1640 medium (Invitrogen, Carlsbad, CA, USA) supplemented with 10% fetal bovine serum (FBS; Sigma-Aldrich, St. Louis, MO, USA).

### Cancer specimens

A total of 24 freshly resected pancreatic cancer samples were collected. All cases were histologically confirmed. No patient was given preoperative radiotherapy or chemotherapy. The clinicopathological characteristics of the patients are summarized in Additional file 1: Table S1. The samples were snap-frozen in liquid nitrogen immediately after resection, and stored at − 80 °C until use.

### Quantitative real-time PCR (qRT-PCR) analysis

Total RNA was extracted from tissue and cell samples using Trizol reagent (Invitrogen). cDNA was synthesized with random primers using the High-capacity cDNA Reverse-Transcription Kit following the manufacturer’s instructions (Applied Biosystems, Foster City, CA, USA). The levels of transcripts were determined by real-time PCR assay with SYBR Green I dye (Qiagen GmbH, Hilden, Germany). PCR primers are as follows: LINC00346 forward, 5′-CACCATGTTGGCCAGGCTGGT-3′; LINC00346 reverse, 5′-GGCCAAAGAGTGACCATCATC-3′; β-actin forward 5′-ATCCACGAAACTACCTTCAACTC-3′; β-actin reverse, 5′-GAGGAGCAATGATCTTGATCTTC-3′. β-Actin was used as a normalizing control. Mature miR-188-3p was detected by the TaqMan MicroRNA Assay, according to the manufacturer’s instructions (Applied Biosystems). U6 small nuclear RNA was used as an internal control. The relative gene expression was determined using the 2^−ΔΔCt^ method [21].

### Plasmids, small interfering RNAs (siRNAs), and transfections

For LINC00346 overexpression studies, a fragment containing the linc00346 sequence (NR_027701.1) was cloned to pcDNA3.1(+) expression vector. Plasmids expressing miR-188-3p, miR-1224-3p, miR-505-5p, and BRD4 were purchased from Hanyu Biomedical Center (Beijing, China). LINC00346-targeting shRNA was synthesized by Sengong Biotech. (Shanghai, China), with the sense sequence as follows: 5′-CCGGAAGCACAGTGGTCTAAAAGTACTCGAGTACTTTTAGACCACTGTGCTTTTTTTG-3′ [13]. For luciferase reporter assays, the *BRD4* 3’-UTR or LINC00346 was cloned into the pMIR-REPORT Luciferase miRNA Expression Reporter Vector (ThermoFisher Scientific, Waltham, MA, USA). Site mutations were generated by PCR using the QuikChange site-directed mutagenesis kit (Stratagen, Santa Clara, CA, USA). All constructs were confirmed by DNA sequencing. siRNA duplexes targeting *BRD4* and nonspecific siRNAs were purchased from Santa Cruz Biotechnology (Santa Cruz, CA, USA).

Transfections were performed using Fugene (Roche Diagnostics, Indianapolis, IN, USA) following the manufacturer’s instructions. For generation of stable cell clones, transfected cells were selected using 600 μg/mL of G418 (Sigma-Aldrich, St. Louis, MO, USA) or 2 μg/mL of puromycin (Sigma-Aldrich).

### Cell proliferation assays

Cells were seeded onto 96-well plates (4 × 10^3^ cells/well) and cultured for 1, 3, and 5 days. Cell viability was assessed using 3-(4,5-dimethylthiazol-2-yl)-2,5-diphenyltetrazolium bromide (MTT; Sigma-Aldrich). Briefly, MTT (5 mg/ml) was added and incubated for 4 h at 37 °C. Dimethyl sulfoxide was added to solubilize the formazan product. Absorbance was measured at 570 nm with a multifunctional microplate reader.

Cell proliferation was also assessed using EdU incorporation assay. In brief, cells were incubated with EdU (50 μM; Beyotime, Haimen, China) for 5 h. After fixation with 4% paraformaldehyde and permeabilization in 1% Triton X-100, the cells were incubated with the staining solution for 30 min in the dark. Nuclei were counterstained with 4′,6-diamidino-2-phenylindole (DAPI; Sigma-Aldrich). EdU-positive cells were examined under a fluorescence microscope.

### Colony formation assay

Cells were plated onto 6-well plates (800 cells/well). The cells were cultured for 10–14 days. Cell were stained with 0.1% crystal violet. The number of colonies was counted under a microscope.

### Animal studies

Female BALB/c nude mice (5 week old) were purchased from the Laboratory Animal Center of the Chinese Academy of Sciences (Shanghai, China). LINC00346-overexpressing and control PANC-1 cells (2 × 10^6^) were subcutaneously injected into nude mice (*n* = 4 for each group). Tumor volume was determined every week for 4 weeks. For assessment of gemcitabine toxicity in vivo, LINC00346-depleted and control PANC-1 cells were subcutaneously inoculated into nude mice (n = 4) and allowed to grow to palpable tumors. The tumor-bearing mice were intraperitoneally injected with vehicle or gemcitabine (Aladdin, Shanghai, China; 100 mg/kg body weight; twice per week) [22]. Tumor size was measured every 3 days after treatment. After the last measurement, mice were sacrificed and tumors were resected and weighed.

### Drug sensitivity assay

To assess the chemosensitivity to gemcitabine, transfected cells were plated onto 96-well plates and treated with different concentrations of gemcitabine (0.1, 1, 10, and 100 μM) for 72 h [23]. Then, the number of viable cells was determined by the MTT assay, and the half maximal inhibitory concentration (IC_50_) was calculated.

### Apoptosis analysis by annexin V-FITC staining

Cells were trypsinized and resuspended in binding buffer containing Annexin V-FITC (BD Biosciences, San Jose, CA, USA) and propidium iodide (PI; BD Biosciences) for 15 min in the dark. Stained cells were analyzed using a flow cytometer (BD Biosciences).

### Cell cycle analysis

For analysis of cell cycle distribution, cells were fixed with ice-cold 70% ethanol and incubated with PI (50 μg/mL) in the presence of RNase A (Sigma-Aldrich) for 30 min. DNA content was analyzed by flow cytometry.

### Caspase-3 activity assay

The activity of caspase-3 was analyzed using the Caspase-3 Colorimetric Assay Kit (Abcam, Cambridge, UK) following the manufacturer’s instructions. In brief, cells were lysed and incubated with the reaction buffer containing DEVD-*p*-NA substrate for 2 h at 37 °C. The absorbance was measured at 405 nm.

### Western blot analysis

Cells were lysed in radioimmunoprecipitation assay buffer supplemented with a protease inhibitor cocktail (Roche Diagnostics). Protein concentrations were measured using the BCA Protein Assay Kit (Pierce, Rockford, IL, USA). Protein samples were resolved by SDS-polyacrylamide gel electrophoresis and transferred to nitrocellulose membranes. The membranes were incubated with anti-p21, anti-P-Chk1, anti-Chk1, anti-P-Chk2, anti-Chk2, anti-BRD4, and anti-β-actin (all from Cell Signaling Technology, Beverly, MA, USA). After washing, the membranes were then incubated with secondary antibodies coupled to horseradish peroxidase for 1 h at room temperature. Protein signals were detected by the Western Lightning Plus ECL kit (PerkinElmer, Waltham, MA, USA) and quantified by densitometry.

### Dual-luciferase reporter assay

293 T cells were seeded onto 24-well plates and transfected with the reporter constructs together with the miR-188-3p-expressing plasmid or empty vector. The pRL-TK plasmid (Promega, Fitchburg, WI, USA) expressing *Renilla* luciferase was co-transfected to control for transfection efficiency. Forty-eight hours after transfection, luciferase activities were measured using the Dual-Luciferase Reporter Assay System (Promega), according to the manufacturer’s instructions. The relative luciferase activity was determined after normalization against *Renilla* luciferase activity.

### RNA-binding protein immunoprecipitation (RIP)

RIP assay was performed as described previously [24]. Briefly, PANC-1 cells were transfected with LINC00346 and miR-188-3p and resuspended in lysis buffer. Cellular lysates were incubated with Protein G sepharose beads conjugated with anti-Ago2 (Abcam) or anti-IgG (Abcam) for 4 h at 4 °C. The immunoprecipitates were treated with DNAse I and proteinase K for 20 min at room temperature. Co-precipitated RNA was recovered and subjected to qRT-PCR analysis.

### Fluorescence in situ hybridization (FISH)

Cy3-labeled LINC00346 and FITC-labeled miR-188-3p probes were purchased from Hanyu Biomedical Center. PANC-1 cells were fixed in 4% formaldehyde and permeabilized with 0.5% TritonX-100. The cells were then hybridized with Cy3- and FITC-labeled probes. Nuclei were stained with DAPI. Images were acquired on a confocal microscope.

### Statistical analysis

All values are reported as mean ± standard deviation and analyzed by the Student’s *t*-test or one-way analysis of variance followed by the post hoc Tukey’s test. Pearson correlation analysis was conducted to determine the relationship between LINC00346 and miR-188-3p levels in pancreatic cancer specimens. Statistical significance was considered at *P* < 0.05.

## Results

### Overexpression of LINC00346 facilitates the growth of pancreatic cancer

**Fig. 1:**
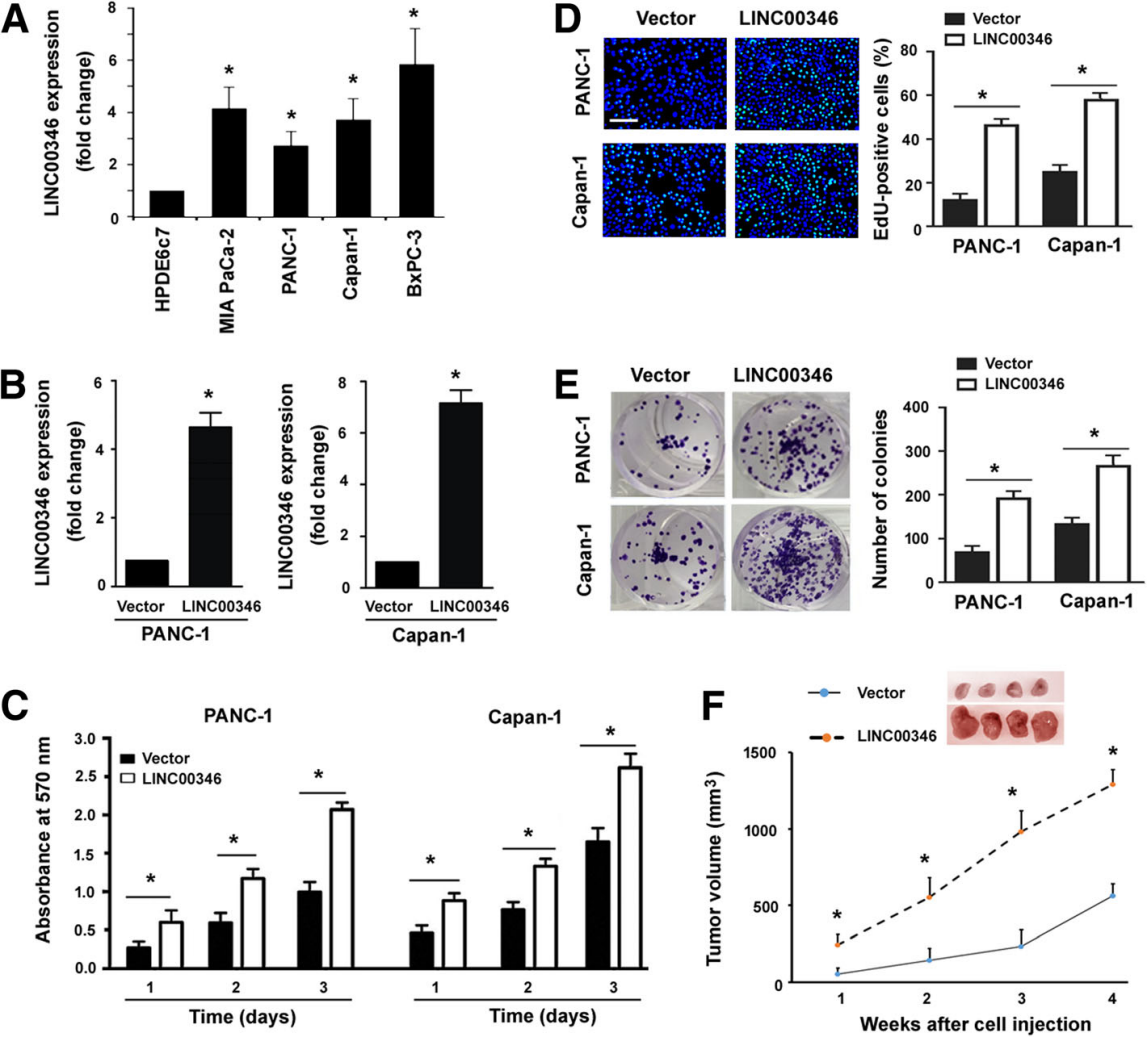
Overexpression of LINC00346 promotes the growth of pancreatic cancer. **a** Measurement of LINC00346 expression levels in pancreatic cancer cells. ^*^P < 0.05 vs. HPDE6c7 pancreatic epithelial cells. **b** Real-time PCR analysis confirmed the upregulation of LINC00346 in PANC-1 and Capan-1 cells transfected with vector or LINC00346-expressing plasmid. **c** MTT assay was performed to assess the proliferation of pancreatic cancer cells transfected with indicated constructs. **d** EdU incorporation assay demonstrated that LINC00346 overexpression increased the fraction of EdU-positive proliferating cells (green). Nuclei stained with DAPI (blue). Scale bar = 100 μm. **e** Pancreatic cancer cells transfected with indicated constructs were subjected to colony formation assay. **f** Xenograft tumor studies. PANC-1 cells transfected with vector or LINC00346-expressing plasmid were subcutaneously injected into nude mice, and tumor growth curves were plotted (*n* = 4). ^*^P < 0.05 vs. the vector group

To investigate the biological relevance of LINC00346 in pancreatic cancer, we performed gain- and loss-of-function studies. Real-time PCR analysis revealed that LINC00346 was expressed at higher levels in pancreatic cancer cells than in HPDE6c7 pancreatic epithelial cells (Fig. 1a). Overexpression of LINC00346 (Fig. 1b) significantly augmented the proliferation of PANC-1 and Capan-1 cells, as determined by MTT assay (Fig. 1c). EdU incorporation assay confirmed that LINC00346 overexpression was associated with a higher percentage of EdU-positive proliferating cells (Fig. 1d). Colony formation assay further demonstrated that LINC00346 overexpression increased the colony formation ability of pancreatic cancer cells (Fig. 1e). Consistent with in vitro findings, LINC00346 overexpression accelerated the growth of PANC-1 cells in xenograft mouse models (Fig. 1f). These results point toward a growth-promoting role for LINC00346 in pancreatic cancer.

### Knockdown of LINC00346 exerts growth-suppressive effects on pancreatic cancer cells

**Fig. 2:**
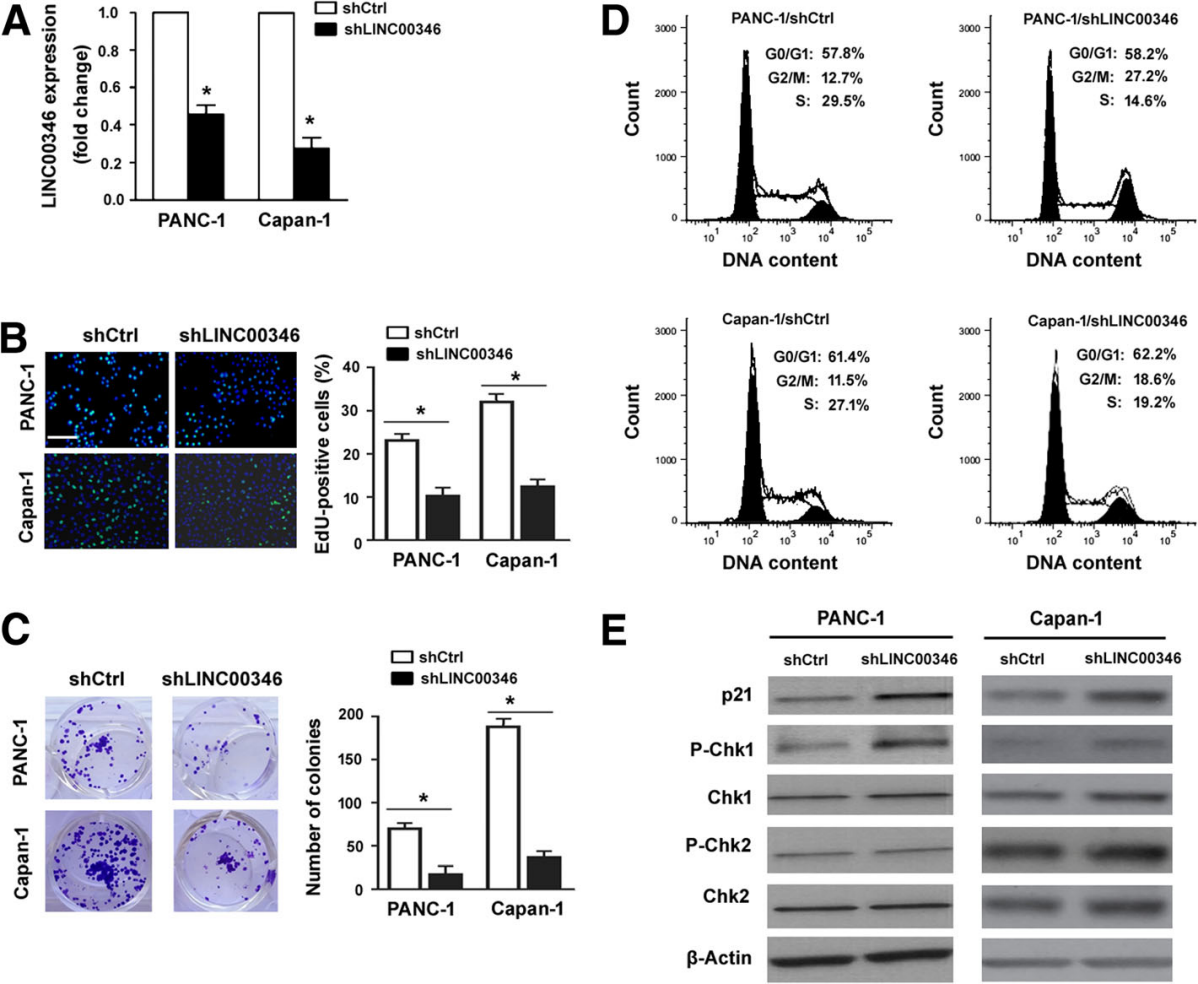
Knockdown of LINC00346 exerts growth-suppressive effects on pancreatic cancer cells. **a** Real-time PCR analysis validated the knockdown of LINC00346 in PANC-1 and Capan-1 cells transfected with control shRNA (shCtrl) or LINC00346-targeting shRNA. **b** Assessment of cell proliferation by EdU incorporation assay. Scale bar = 100 μm. **c** Colony formation assay was used to determine the colony-forming ability. ^*^P < 0.05 vs. the shCtrl group. **d** Cell cycle distribution by flow cytometry after PI staining. Numbers indicate mean ± s.d. percentage of cells in each phase of the cell cycle. **e** Western blot analysis of indicated proteins

Next, we performed LINC00346 knockdown experiments using specific shRNA. Transfection with LINC00346-targeting shRNA efficiently suppressed the endogenous expression of LINC00346 in PANC-1 and Capan-1 cells (Fig. 2a). Depletion of LINC00346 remarkably decreased the proliferation (Fig. 2b) and colony formation (Fig. 2c) of pancreatic cancer cells. Analysis of cell cycle distribution demonstrated that the proportion of the S-phase cells was significantly decreased and that of the G2/M-phase cells was increased after LINC00346 depletion (Fig. 2d), indicating a cell cycle arrest at the G2/M phase. The cell cycle regulatory proteins p21 and phosphorylated Chk1 were increased in LINC00346-depleted cells (Fig. 2e). These results provides more insight into the mechanism for the proliferation defects observed in LINC00346-depleted pancreatic cancer cells.

### Depletion of LINC00346 enhances gemcitabine sensitivity in pancreatic cancer cells

**Fig. 3:**
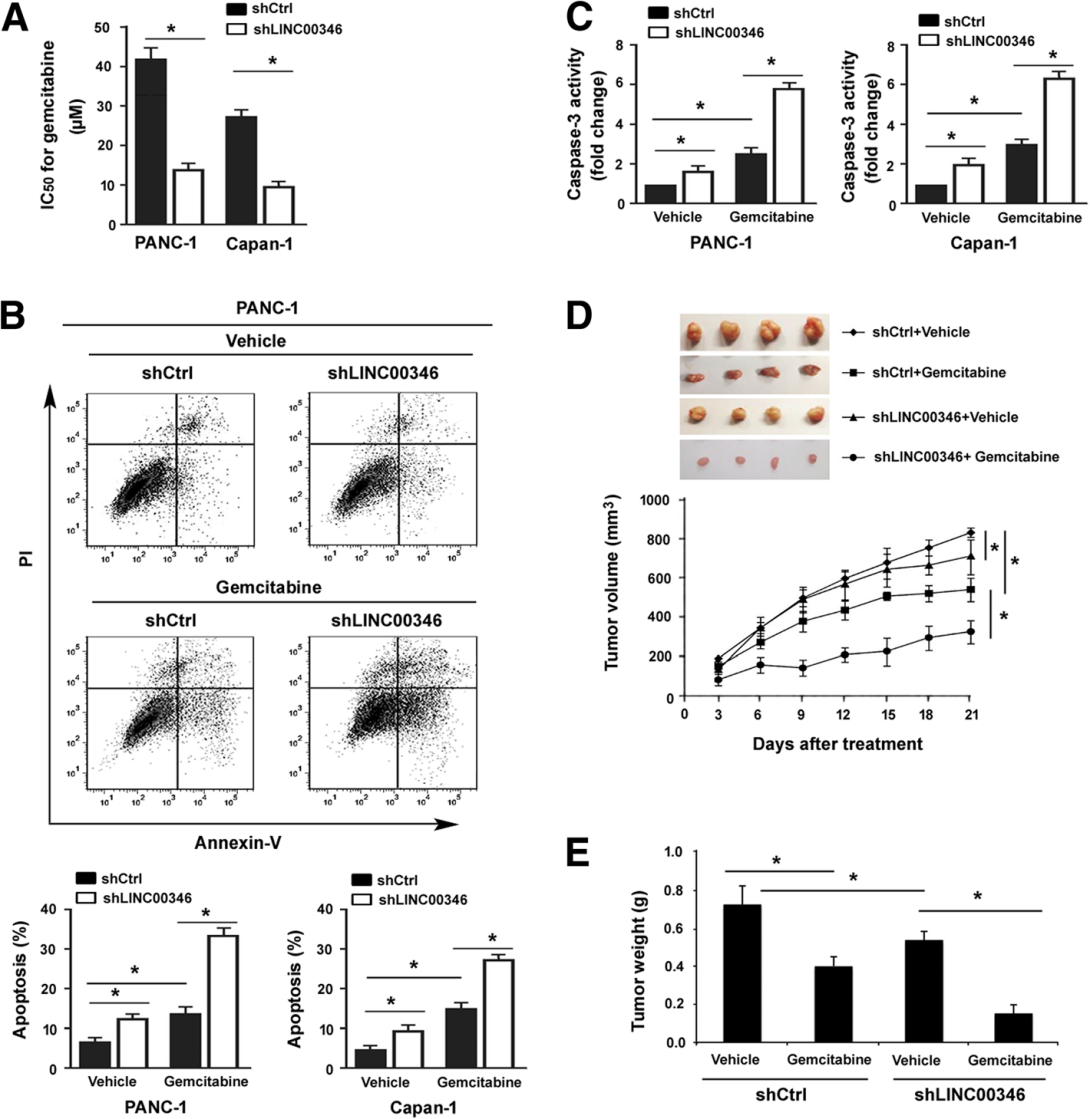
Depletion of LINC00346 enhances gemcitabine sensitivity in pancreatic cancer cells. **a** The IC50 value for gemcitabine was significantly lower in LINC00346-depleted cells than that in control cells. **b** Flow cytometric analysis of apoptosis in PANC-1 and Capan-1 cells after indicated treatments. *Upper*, representative dot plots of Annexin-V/PI staining for apoptosis in PANC-1 cells. Here, 40 μM gemcitabine was used. **c** Measurement of caspase-3 activity in the cells treated as in (**b**). **d** LINC00346-depleted and control PANC-1 cells were subcutaneously injected into nude mice and then treated with gemcitabine, and tumor growth curves were plotted (n = 4). Inserts are representative tumors from 4 mice. **e** Tumor weight was determined for each group. ^*^P < 0.05

Next, we asked whether LINC00346 can influence the sensitivity of pancreatic cancer cells to gemcitabine. To address this issue, we exposed LINC00346-depleted and control PANC-1 and Capan-1 cells to different concentrations of gemcitabine for 48 h and examined the changes in cell viability. When LINC00346 was knocked down, PANC-1 and Capan-1 cells showed more susceptibility to gemcitabine, with a significant decline in the IC_50_ value for gemcitabine (Fig. 3a). Furthermore, gemcitabine-induced apoptosis was dramatically reinforced by silencing of LINC00346 (Fig. 3b), which was accompanied by significantly higher caspase-3 activity (Fig. 3c). We also investigated the effect of inhibition of LINC00346 on chemotherapeutic response in vivo. LINC00346-depleted and control PANC-1 cells were subcutaneously injected into nude mice and then treated with gemcitabine. We found that gemcitabine treatment significantly restrained the growth of xenograft tumors from LINC00346-depleted PANC-1 cells, relative to control tumors (Fig. 3d and e). Collectively, these findings suggest the targeting of LINC00346 is beneficial in improving the chemotherapeutic sensitivity of pancreatic cancer cells to gemcitabine.

### LINC00346 interacts with miR-188-3p in pancreatic cancer

**Fig. 4:**
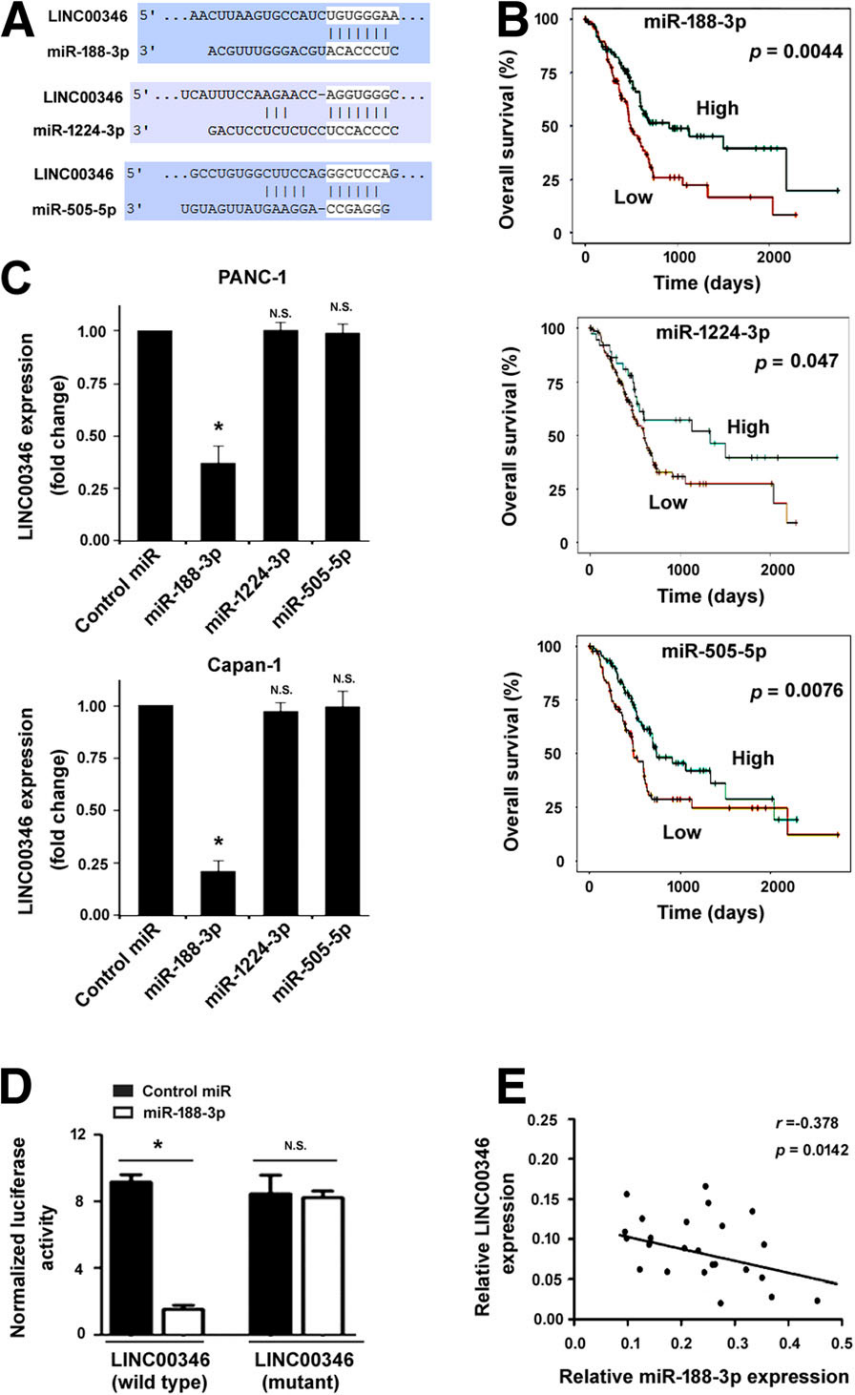
LINC00346 interacts with miR-188-3p in pancreatic cancer. **a** Prediction of target sites for miR-188-3p, miR-1224-3p, and miR-505-5p in LINC00346 based on the online program (http://www.targetscan.org/vert_71/). **b** Kaplan–Meier survival analysis of miR-188-3p, miR-1224-3p, and miR-505-5p expression in pancreatic cancer based on TCGA datasets (http://starbase.sysu.edu.cn/panCancer.php). **c** Determination of the effect of overexpression of miRs on the expression of LINC00346 in pancreatic cancer cells. ^*^P < 0.05 vs. control miR; n.s. indicates no significance. **d** Luciferase reporter assay showed that miR-188-3p overexpression significantly suppressed the activity of the reporter containing wild-type LINC00346. ^*^P < 0.05; n.s. indicates no significance. **e** The expression of LINC00346 and miR-188-3p in pancreatic cancer specimens (*n* = 24)

Mounting evidence has indicated the close link between lncRNAs and miRNAs in the regulation of biological processes [9, 10]. To explore the mechanism by which LINC00346 affects pancreatic cancer growth and chemosensitivity, we used bioinformatic analysis to search for miRNAs that can interact with LINC00346. The results showed that LINC00346 harbors putative target sites for many miRNAs, especially miR-188-3p, miR-1224-3p, and miR-505-5p (Fig. 4a), whose expression tended to be associated with prognosis of pancreatic cancer based on TCGA datasets (Fig. 4b). Ectopic expression of miR-188-3p but not miR-1224-3p or miR-505-5p led to a significant inhibition of LINC00346 expression in pancreatic cancer cells (Fig. 4c). These data suggest that LINC00346 may be a target of miR-188-3p. Dual-luciferase reporter assays confirmed that miR-188-3p overexpression significantly suppressed the activity of the reporter containing wild-type LINC00346 (Fig. 4d). In addition, there was a negative correlation between the expression of LINC00346 and miR-188-3p in pancreatic cancer specimens (*r* = − 0.378, *P* = 0.0142; Fig. 4e).

### LINC00346 rescues the inhibitory effect of miR-188-3p on pancreatic cancer cells

**Fig. 5:**
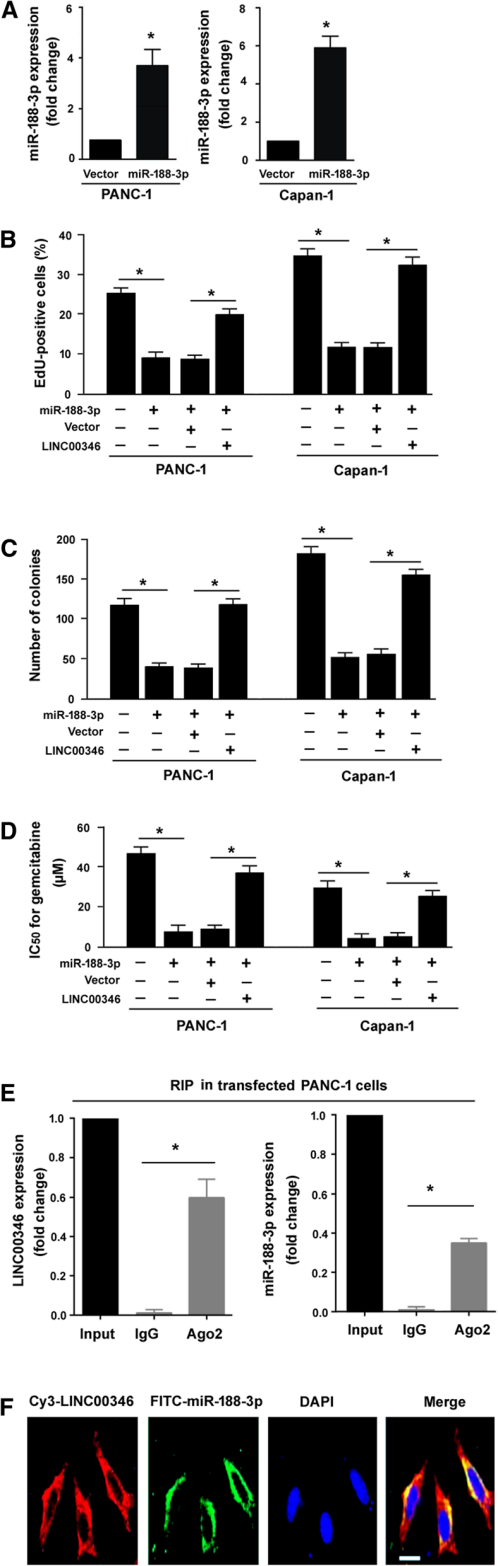
LINC00346 rescues the inhibitory effect of miR-188-3p on pancreatic cancer cells. **a** Real-time PCR analysis confirmed the upregulation of miR-188-3p in PANC-1 and Capan-1 cells transfected with vector or miR-188-3p-expressing plasmid. **b** Cell proliferation determined by EdU incorporation assay. **c** Colony formation assay in cells transfected with indicated constructs. **d** miR-188-3p overexpression significantly enhanced gemcitabine cytotoxicity, which was abolished by overexpression of LINC00346. **e** Ago2-based RIP assay performed in PANC-1 cells co-transfected with LINC00346 and miR-188-3p. ^*^P < 0.05. **f** Analysis of the intracellular distribution of LINC00346 and miR-188-3p in PANC-1 cells by FISH. Scale bar = 20 μm. Nuclei were counterstained with DAPI (blue)

Next, we investigated the role of miR-188-3p in pancreatic cancer. Ectopic expression of miR-188-3p (Fig. 5a) significantly repressed the growth (Fig. 5b) and colony formation (Fig. 5c) of PANC-1 and Capan-1 cells. Moreover, miR-188-3p overexpression significantly enhanced gemcitabine cytotoxicity to PANC-1 and Capan-1 cells (Fig. 5d). These data indicate miR-188-3p as a tumor suppressor in pancreatic cancer.

Next, we checked whether LINC00346 can interfere with the action of miR-188-3p in pancreatic cancer development. Of note, enforced expression of LINC00346 remarkably restored the proliferation (Fig. 5b) and colony formation (Fig. 5c) of miR-188-3p-overexpressing PANC-1 and Capan-1 cells. Moreover, the chemosensitizing effect of miR-188-3p was significantly antagonized by overexpression of LINC00346 (Fig. 5d). These results suggest that LINC00346 acts as a competitive RNA for miR-188-3p. In support of this hypothesis, both LINC00346 and miR-188-3p were detected in Ago2 immunoprecipitates from pancreatic cancer cells (Fig. 5e). We also investigated the intracellular distribution of LINC00346 and miR-188-3p in pancreatic cancer cells by FISH. As shown in Fig. 5f, LINC00346 and miR-188-3p were co-localized in the cytoplasm of PANC-1 cells.

### LINC00346 counteracts miR-188-3p to promote BRD4 expression

**Fig. 6:**
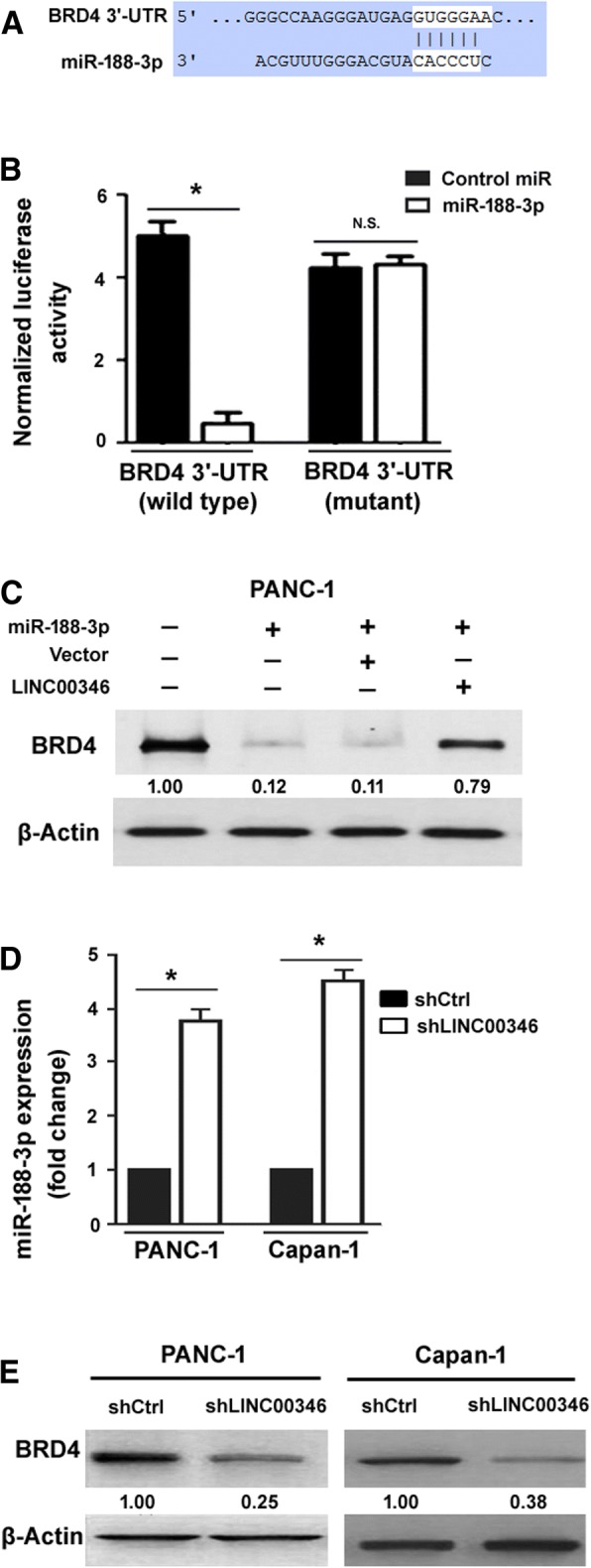
LINC00346 counteracts miR-188-3p to promote BRD4 expression. **a** Prediction of a target site for miR-188-3p in the 3’-UTR of *BRD4* mRNA based on the online program (http://www.targetscan.org/vert_71/). **b** Luciferase reporter assay confirmed that the reporter containing the 3’-UTR of BRD4 was repressed by overexpression of miR-188-3p. ^*^P < 0.05; n.s. indicates no significance. **c** Western blot analysis of BRD4 protein in PANC-1 cells transfected with indicated constructs. Numbers indicate fold change in BRD4 protein levels relative to control cells. **d** Real-time PCR analysis of miR-188-3p levels in PANC-1 and Capan-1 cells transfected with control shRNA (shCtrl) or LINC00346-targeting shRNA (shLINC00346). ^*^P < 0.05. **e** Western blot analysis of BRD4 protein in PANC-1 and Capan-1 cells transfected with indicated constructs

Next, we attempted to identify the direct targets of miR-188-3p. Bioinformatic analysis predicted a target site for miR-188-3p in the 3’-UTR of *BRD4* mRNA (Fig. 6a). Luciferase reporter assay confirmed that the reporter containing the 3’-UTR of *BRD4* was repressed by overexpression of miR-188-3p (Fig. 6b). Apart from BRD4, 5 other candidate targets (i.e., MDM4, NKX6–1, NEK3, RAB26, and PDX1) were chosen for validation by luciferase reporter assays. It was found that the reporter harboring the 3’-UTR of *MDM4*, *NKX6–1*, *NEK3*, *RAB26*, or *PDX1* was not affected by miR-188-3p overexpression (data not shown). The endogenous level of BRD4 in pancreatic cancer cells was also decreased by miR-188-3p overexpression (Fig. 6c). Since BRD4 and LINC00346 shared the same response element complementary to the seed sequence of miR-188-3p (Fig. 4a and Fig. 6a), we tested whether overexpression of LINC00346 can prevent miR-188-3p targeting to BRD4 mRNA. When LINC00346 was co-expressed, miR-188-3p-mediated downregulation of BRD4 was markedly reversed (Fig. 6c). In addition, knockdown of LINC00346 resulted in an elevation of miR-188-3p (Fig. 6d) and reduction of BRD4 protein (Fig. 6e) in PANC-1 and Capan-1 cells. Taken together, LINC00346 has the ability to mask BRD4 from miR-188-3p-dependent repression in pancreatic cancer.

### BRD4 mediates the growth-promoting activity of LINC00346

**Fig. 7:**
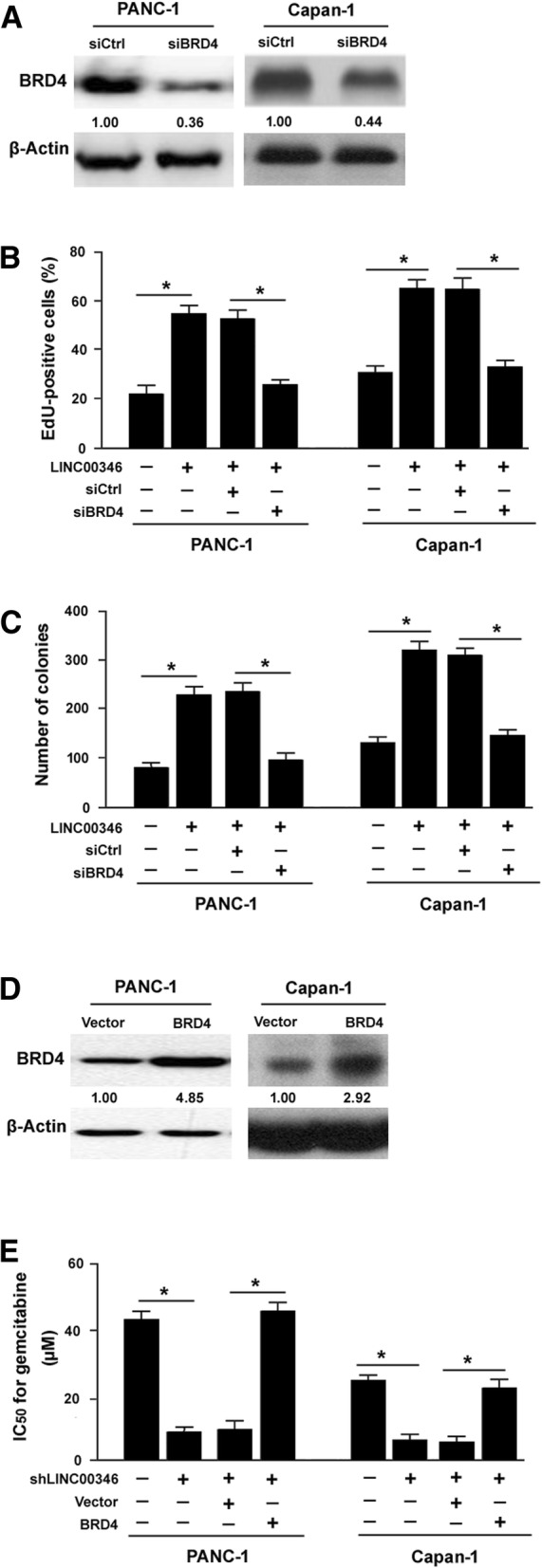
BRD4 mediates the growth-promoting activity of LINC00346. **a** Western blot analysis of BRD4 protein levels in pancreatic cancer cells transfected with control siRNA (siCtrl) or BRD4-targeting siRNA (siBRD4). Numbers indicate fold change in BRD4 protein levels relative to control cells. **b** and **c** PANC-1 and Capan-1 cells transfected with indicated constructs were tested for proliferation (**b**) and colony formation (**c**). ^*^P < 0.05. **d** Western blot analysis of BRD4 protein levels in pancreatic cancer cells transfected with indicated constructs. **e** LINC00346 depletion-induced gemcitabine sensitivity was reversed by overexpression of BRD4. ^*^P < 0.05

To check whether BRD4 is involved in LINC00346-mediated oncogenic effects, we performed BRD4 knockdown experiments. The efficiency of BRD4 knockdown was confirmed by Western blot analysis (Fig. 7a). We found that silencing of BRD4 significantly inhibited the proliferation (Fig. 7b) and colony formation (Fig. 7c) of pancreatic cancer cells stably expressed LINC00346. To check whether BRD4 is involved in LINC00346 inhibition-induced chemosensitizing effect, we performed rescue experiments by ectopically expressing BRD4 (Fig. 7dd). We found that overexpression of BRD4 almost completely blocked the chemosensitizing effect on pancreatic cancer cells induced by LINC00346 depletion (Fig. 7e). These results support the hypothesis that BRD4 is implicated in LINC00346-induced growth and gemcitabine resistance in pancreatic cancer.

## Discussion

It has been previously reported that increased expression of LINC00346 is associated with shorter overall survival of pancreatic cancer patients [14]. Moreover, LINC00346 shows the capacity to promote aggressive phenotype in lung cancer and bladder cancer cells [12, 13]. These indications encouraged us to explore the biological function of LINC00346 in pancreatic cancer. We found that LINC00346 is highly expressed in a panel of pancreatic cancer cell lines, which is in line with clinical observations [14]. Of note, our data demonstrate that ectopic expression of LINC00346 enhances the proliferation and colony formation of pancreatic cancer cells, whereas knockdown of LINC00346 yields an opposite effect. In vivo data further indicate that LINC00346 overexpression augments pancreatic cancer xenograft growth. These data validate an important role for LINC00346 in pancreatic cancer growth.

EdU incorporation assays reveal that depletion of LINC00346 significantly reduced the percentage of EdU-positive cells, suggesting a decrease in DNA replication. Furthermore, analysis of cell cycle progression indicate that knockdown of LINC00346 arrests PANC-1 and Capan-1 cells at the G2/M phase. Our data support an involvement of LINC00346 in cell cycle progression. Biochemically, LINC00346 knockdown leads to an increase in the cell-cycle regulators p21 and phosphorylated Chk1. p21 is a well-established cyclin-dependent kinase inhibitor and causes a negative regulation of cell-cycle progression [25]. There is evidence that p21 can sustain cell-cycle arrest at the G2/M phase by inducing degradation of cyclin B1 [26]. Chk1 is a serine/threonine-specific protein kinase. Similar to p21, Chk1 also affects cell-cycle progression at the G2/M phase [27]. Therefore, the G2/M cell-cycle arrest observed in LINC00346-depleted pancreatic cancer cells may be a consequence of induction of p21 expression and Chk1 phosphorylation.

Several lncRNAs such as Linc-ROR, AB209630, and PVT1 have been reported to be involved in gemcitabine resistance in pancreatic cancer [11, 28, 29]. In this study, we identify a novel lncRNA regulator of gemcitabine sensitivity and indicate that LINC00346 depletion sensitizes pancreatic cancer cells to gemcitabine. We found that knockdown of LINC00346 enhances gemcitabine-induced apoptosis in pancreatic cancer cells. Furthermore, the activity of caspase-3 is remarkably increased in LINC00346-depleted cells after gemcitabine treatment, suggesting the involvement of caspase-3 in the apoptosis process. In vivo studies further confirm that xenograft tumors from LINC00346-depleted Capan-1 cells display more susceptibility to gemcitabine. Overall, we provide first evidence for the ability of LINC00346 to influence the gemcitabine sensitivity of pancreatic cancer cells.

Mechanistically, LINC00346 can be negatively regulated by miR-188-3p. We found that overexpression of miR-188-3p significantly inhibits the expression of LINC00346 in pancreatic cancer cells. Luciferase reporter assay validated the presence of a functional response element specific for miR-188-3p in LINC00346. Biochemically, both LINC00346 and miR-188-3p were present in Ago2 immunoprecipitates. Moreover, LINC00346 and miR-188-3p were co-localized in pancreatic cancer cells. Although miR-188-3p has a pro-metastatic activity in colorectal cancer [30], an opposite effect is noted in pancreatic cancer. We demonstrate that overexpression of miR-188-3p causes growth reduction and enhances gemcitabine cytotoxicity in pancreatic cancer cells. Considering that LINC00346 and miR-188-3p exert opposite effects against pancreatic cancer cells, we hypothesized that the oncogenic activity of LINC00346 may be ascribed to the interaction with miR-188-3p. In support of this hypothesis, we showed that overexpression of LINC00346 reverses the growth-suppressive and chemosensitizing effects evoked by miR-188-3p. Clinical evidence further reveals a negative correlation between the expression of LINC00346 and miR-188-3p in pancreatic cancer specimens. Taken together, LINC00346 confers aggressive phenotype by sponging miR-188-3p.

Identification of functional target genes is of importance in exploring the mechanism for miR action [31]. Our findings demonstrate that the 3’-UTR of *BRD4* mRNA harbors a functional response element for miR-188-3p. Moreover, ectopic expression of miR-188-3p significantly inhibits the expression of BRD4 in pancreatic cancer cells. Therefore, BRD4 serves as a downstream target of miR-188-3p. Most interestingly, LINC00346 overexpression abrogates miR-188-3p-mediated repression of BRD4 in pancreatic cancer cells. Since BRD4 is involved in the growth and gemcitabine chemoresistance of pancreatic cancer cells [19, 20], we suggest that LINC00346 promotes aggressiveness of pancreatic cancer cells by sponging miR-188-3p and inducing BRD4. This argument is supported by the finding that knockdown of BRD4 significantly prevents LINC00346-induced pancreatic cancer growth.

## Conclusion

LINC00346 contributes to pancreatic cancer growth and gemcitabine chemoresistance. The tumor-supporting role of LINC00346 could in part be explained by antagonization of miR-188-3p and derepression of BRD4 expression. Therefore, LINC00346 represents a potential target for improving chemotherapeutic efficacy for pancreatic cancer.

## Additional file

Additional file 1: **Table S1.** Characteristics of the patients included in this study. (DOC 24 kb)

## Abbreviations

BRD4: Bromodomain-containing protein 4
ceRNAs: Competing endogenous RNAs
lncRNAs: Long non-coding RNAs
miRs: microRNAs
qRT-PCR: Quantitative real-time PCR
RIP: RNA-Binding protein immunoprecipitation
siRNAs: small interfering RNAs
