# Supplementary material for: Long non-coding RNA LINC00346 promotes pancreatic cancer growth and gemcitabine resistance by sponging miR-188-3p to derepress BRD4 expression
Source: J Exp Clin Cancer Res. 2019 Feb 6;38:60. doi: 10.1186/s13046-019-1055-9 (PMC6366022; doi:10.1186/s13046-019-1055-9)
Supplement: Supplementary file 1 — Table S1. Characteristics of the patients included in this study. (DOC 24 kb) [file 13046_2019_1055_MOESM1_ESM.doc]

**Supplementary Table S1. Characteristics of the patients included in this study.**

| **Features** | **Number** |
| --- | --- |
| Age, years |  |
| <60 | 19 |
| ≥60 | 5 |
| Gender |  |
| Male | 17 |
| Female | 7 |
| Tumor size, cm |  |
| <3 | 9 |
| ≥3 | 15 |
| Histological grade |  |
| Well/moderate | 11 |
| Poor | 13 |
| TNM stage |  |
| I-II | 10 |
| III-IV | 14 |
